# The Role of Neutrophils in the Induction of Specific Th1 and Th17 during Vaccination against Tuberculosis

**DOI:** 10.3389/fmicb.2016.00898

**Published:** 2016-06-10

**Authors:** Monalisa M. Trentini, Fábio M. de Oliveira, André Kipnis, Ana P. Junqueira-Kipnis

**Affiliations:** Laboratory of Immunopathology of Infectious Disease, Department of Microbiology, Immunology, Parasitology and Pathology, Institute of Tropical Disease and Public Health, Federal University of GoiásGoiânia, Brazil

**Keywords:** tuberculosis vaccines, Th1/Th17 responses, neutrophils

## Abstract

*Mycobacterium tuberculosis* causes tuberculosis (TB), a disease that killed more than 1.5 million people worldwide in 2014, and the Bacillus Calmette Guérin (BCG) vaccine is the only currently available vaccine against TB. However, it does not protect adults. Th1 and Th17 cells are crucial for TB control, as well as the neutrophils that are directly involved in DC trafficking to the draining lymph nodes and the activation of T lymphocytes during infection. Although several studies have shown the importance of neutrophils during *M. tuberculosis* infection, none have shown its role in the development of a specific response to a vaccine. The vaccine mc^2^-CMX was shown to protect mice against *M. tuberculosis* challenge, mainly due to specific Th1 and Th17 cells. This study evaluated the importance of neutrophils in the generation of the Th1- and Th17-specific responses elicited by this vaccine. The vaccine injection induced a neutrophil rich lesion with a necrotic central area. The IL-17 KO mice did not generate vaccine-specific Th1 cells. The vaccinated IL-22 KO mice exhibited Th1- and Th17-specific responses. Neutrophil depletion during vaccination abrogated the induction of Th1-specific responses and prohibited the bacterial load reduction observed in the vaccinated animals. The results show, for the first time, the role of neutrophils in the generation of specific Th1 and Th17 cells in response to a tuberculosis vaccine.

## Introduction

The only available prevention against tuberculosis (TB) is the *Mycobacterium bovis* BCG vaccine, which is effective against the miliary and meningitis forms of TB in children; however, its efficacy varies greatly among adults, and, as result, TB is the second leading cause of death by an infectious agent worldwide (Kozak and Behr, 2011; World Health Organization [WHO], 2015). Classically, Th1 cells (IFN-γ-, TNF-α-, and IL-12-producing T cells) play an important role in TB control (Cooper and Khader, 2008). For many years, it was accepted that IFN-γ production by T cells was the most important and effective response in the control of TB (Flynn and Chan, 2001). Nevertheless, new studies have shown that, in addition to IFN-γ, the production of other types of cytokines is essential for the generation of an efficient protective response (Gopal et al., 2013). Additionally, CD8^+^ T cells, both in humans and animals, respond to *M. tuberculosis* infection (Lin and Flynn, 2015) being of great importance during the chronic stage of infection. Recently, Booty et al. (2016), showed that IL-12 is crucial for CD8^+^ T cell priming as is IFN-γ to maintain the effector and memory cell subsets during *M. tuberculosis* infection.

For instance, some of the vaccines against TB that are currently undergoing clinical trials have shown an important role for the Th17 cells in the induction of protection (Desel et al., 2011; Hwang et al., 2011). Th17 cells secrete IL-17, IL-22 and IL-21 (Fouser et al., 2008). IL-22, although early induced during *M. tuberculosis* infection, is not crucial to induce Th1 cells, thus was not associated to protection (Behrends et al., 2013). The role of IL-17 in protection against *M. tuberculosis* infection may be related to its role in the induction of Th1 cells (Weaver and Murphy, 2007; Kolls and Khader, 2010). IL-17 is known to activate and recruit neutrophils (Ye et al., 2001; Happel et al., 2005). Additionally, Monin et al. (2015) showed that this cytokine favors the recall of vaccine-specific memory T cells in response to *M. tuberculosis* challenge through an IL-23-dependent mechanism. Recently, it was showed that neutrophils when activated by IL-23 also produce IL-17 and IL-22 (Chen et al., 2016).

Neutrophils are indispensable for the first line of defense against microorganisms (Ye et al., 2001; Torrado and Cooper, 2010), as they can activate different cell types, such as macrophages, dendritic cells (DC), natural killer (NK) cells and B lymphocytes, and secrete cytokines, such as IL-1β (Morel et al., 2008; Cho et al., 2012). Furthermore, Blomgran and Ernst (2011) demonstrated that lung neutrophils are directly involved in DC trafficking to the draining lymph nodes and in the activation of T lymphocytes in a mouse model of TB infection. Both CD4^+^ and CD8^+^ T cells can be primed to *M. tuberculosis* antigens aided by DC crosspriming of engulfing apoptotic bodies from neutrophils (Behar, 2013).

Considering that neutrophils are important in the host–pathogen interaction, and IL-17 and IL-22 are involved in both innate and specific immune responses to *M. tuberculosis*, we addressed the role of neutrophils in the induction of specific Th1 and Th17 cells in response to vaccination against TB and the subsequent protective outcome. To investigate this, we used a vaccine that is capable of inducing Th1- and Th17-specific T cell responses and is protective against TB (Junqueira-Kipnis et al., 2013).

## Materials and Methods

### Vaccine and rCMX Preparation

The protocols to prepare the vaccine as previously described (Junqueira-Kipnis et al., 2013), were strictly followed. Briefly, the mc^2^-CMX vaccine was diluted to 1 × 10^8^ CFU/mL in PBS with 0.05% Tween 80 (PBST). rCMX was obtained as previously described (de Sousa et al., 2012). After protein purification, lipopolysaccharide (LPS) was removed using the ToxinEraser^TM^ Endotoxin Removal Kit (GenScript).

### Animals

Eight-week-old C57BL/6, IL-22 gene knockout (KO) and IL-17ra KO mice, all from C57BL/6 background (originally from Jackson Laboratory) were donated by Dr. João Santana da Silva from Universidade de São Paulo and were housed and bred in micro isolators adapted to HEPA filtered racks with 12 h light/dark cycles, temperature ranging from 20 to 24°C, and 50% humidity at the animal facilities of the Institute of Tropical Disease and Public Health. The procedures used in this study were approved by the Ethical Committee on animal use of Federal University of Goiás [Comitê de Ética no Uso de Animais da Universidade Federal de Goiás (Protocol number: 027/14)].

### Vaccination Design

The C57BL/6 (*n* = 12), IL-17 KO (*n* = 12), and IL-22 KO (*n* = 12) mice were subcutaneously vaccinated with 100 μL (10^7^ CFU) of mc^2^-CMX (*M. smegmatis* expressing CMX). The second dose was administered 15 days later at the inflammatory spot generated by the first immunization. The controls from each mouse strain were vaccinated with PBST (*n* = 12). At 15 days after the last vaccination, five animals from each group were euthanized by cervical dislocation by a trained veterinarian, and the vaccine skin lesions and spleens were collected. The remaining animals were intravenously challenged with *M. tuberculosis* strain H37Rv (10^5^ CFU) 30 days after the booster vaccination.

### *M. tuberculosis* Infection

Cultures of *M. tuberculosis* (H37Rv) in 7H9 containing 0.05% Tween 80 and OADC were grown to mid-log phase, and aliquots were frozen in 20% glycerol at -80°C. The concentration of the mycobacteria lot was determined 30 days later by plating and counting the CFUs from representative frozen vials. On the day of the challenge, aliquots were thawed, the concentration was adjusted to 10^6^ CFU/mL, and 100 μL were intravenously injected into the lateral tail vein. One day after infection, one animal from each group was euthanized, and the lungs were homogenized and plated on 7H11 supplemented with OADC media to determine the initial bacterial load. The average load of bacteria in the lungs at day one was 3.4 × 10^4^ CFU.

### *M. tuberculosis* Load Quantification

Thirty days after the challenge, the animals were euthanized and the right cranial and medial lung lobes were homogenized to verify the bacterial load as described above.

### Specific CD4 T Immune Response to CMX

The specific immune response induced by the vaccination was tested before the challenge (15 days after the booster) and 30 days after the *M. tuberculosis* infection. Six animals from each group were euthanized by cervical dislocation and the spleens were removed to assess the specific immune responses. The single cell suspension obtained after passage through a 70 μm strainer (BD Biosciences) was treated with lysis buffer (0.15 M NH_4_Cl and 10 mM KHCO_3_). After washing with RPMI, the concentration of the suspension was adjusted to 1 × 10^6^/mL, and 200 μL were plated in 96-well plates (Cell Wells^TM^). The cells were treated with ConA (1 μg/mL), rCMX (10 μg/mL) or medium and cultured for 4 h at 37°C in a CO_2_ incubator. Then, monensin (eBioscience, 3 μM) was added, and the cells were incubated for an additional 4 h. The plates were centrifuged and the cells were incubated with a FITC-conjugated anti-CD4 antibody (BD PharMingen) for 30 min. Then, the cells were washed, fixed, and permeabilized using the BD Cytofix/Cytoperm kit. The cells were further incubated for 30 min with a PercP-conjugated anti-IL-17 antibody (eBioscience) and an PE-conjugated anti-IFN-γ antibody (eBioscience). The cell suspensions were acquired using a BD Biosciences FACSVerse^TM^ and analyzed with the FlowJo 9.0 Software. A total of 100,000 events or at least 20,000 lymphocytes were acquired per sample. The specific immune response to CMX was obtained by subtracting the percentage of IFN-γ^+^ or IL-17^+^ non-stimulated cells from the percentage of IFN-γ^+^ or IL-17^+^ cells stimulated with CMX. To calculate the total number of CD4 T cells expressing IFN-γ or IL-17, the above percentage differences were multiplied by the total number of splenocytes obtained from each animal. The results were presented as average and standard deviation of each treatment group. Responsiveness of the cell suspensions was checked by Con-A stimulation controls.

### Neutrophil Evaluation

The neutrophils from the vaccinated animals were evaluated 30 days after the challenge with *M. tuberculosis*. The splenocytes were obtained as described above. The cells were incubated for 30 min with APC-conjugated anti-GR1 (BD PharMingen), PercP-conjugated anti-CD14, PE-conjugated anti-CD11c and FITC-conjugated anti-CD11b antibodies (eBioscience). Then, the cells were washed with PBS and fixed with paraformaldehyde using Perm Fix (BD Cytofix). A total of 100,000 events were acquired using BD Biosciences FACSVerse^TM^ and analyzed with the FlowJo 9.0 Software. The neutrophils percentage was determined evaluating singlets that were GR-1^+^, CD11c^-^, and CD14^-^. The total number of neutrophils was obtained multiplying the percentage of neutrophils by the total number of splenocytes from each animal.

### Neutrophil Depletion

Three groups of C57BL/6 mice were treated with 200 μg of anti-mouse Ly6G antibodies (BioXcell; clone 1A8), Rat IgG2a isotype control (BioXcell; clone 2A3), or not treated (PBS control), according to the protocol described (Kozakiewicz et al., 2013). The animals in each of these groups (*n* = 12 mice per group) were equally subdivided into two groups: one that was vaccinated with mc^2^-CMX (10^7^ CFU/animal) and a control PBST vaccinated group. Therefore the experiments consisted of six groups (*n* = 6): Ly6G depleted and vaccinated; Ly6G depleted not vaccinated; isotype control treated and vaccinated; isotype control treated not vaccinated; not treated and vaccinated (vaccine control); and the group not treated nor vaccinated (PBS control). The depletion started 1 day before the vaccination scheme and was maintained for 31 days. At the end of the treatment, three animals from each experimental group were euthanized, and the lungs and spleens were collected and evaluated by flow cytometry. The spleen cells were obtained as described above, and the lung cells were obtained as described (Junqueira-Kipnis et al., 2013). The remaining animals (*n* = 3 for each experimental group) were infected with *M. tuberculosis* (10^5^ CFU/mouse) 46 days after the beginning of the treatment. Thirty days after the challenge, the animals were euthanized, and the lungs were evaluated by flow cytometry. The right cranial and medial lung lobes were homogenized to verify the bacterial load as described above. The experiment was repeated twice.

### Tissue Damage Evaluation

The tissues from the vaccination site and the right caudal lung lobes were collected and fixed in 10% paraformaldehyde. Five-micrometer-thick fixed tissues were placed on slides, stained with hematoxylin and eosin (H&E), and evaluated using an optical microscope (Axioscope A1; Carl Zeiss). The slides were evaluated by a certified Pathologist from Faculdade de Odontologia-Universidade Federal de Goiás blinded to original section sources. Scores were determined based on the area with lesions relative to the area of the total visual field using AxioVision microscopy Software. The results are presented as the percentage of area with lesions. Three different fields were evaluated per slide for each animal from each group (da Costa et al., 2014).

### Statistical Analysis

The data were imported to Excel (version 14.3.4, 2011) and analyzed using Graphpad Software 4.0. The comparisons between the groups were evaluated by ANOVA followed by the Mann–Whitney *U* test. *p* < 0.05 was considered statistically significant. For depletions assay the groups were using Tukey’s multiple comparison test.

## Results

### Subcutaneous Vaccination with *M. smegmatis* mc^2^-CMX Induces Local Abscess Formation

**FIGURE 1:**
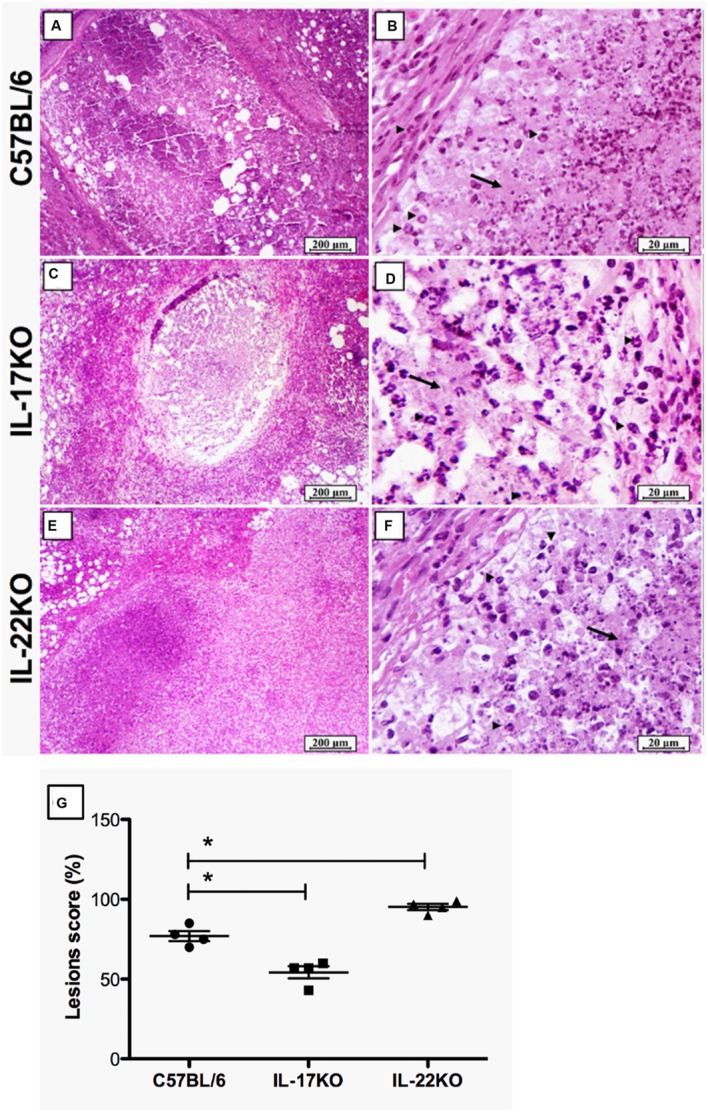
**Vaccination induces abscess at the injection site.** The C57BL/6, IL-17 KO, and IL-22 KO mice were subcutaneously injected with the *Mycobacterium smegmatis*-CMX (mc^2^-CMX) vaccine, and the skin lesion at the vaccination site was evaluated by H&E staining 15 days after immunization. The mc^2^-CMX vaccine induces abscess with higher influx of neutrophils (arrow heads) and central necrosis (arrow) in the C57BL/6 **(A,B)**, IL-22 KO **(C,D)**, and IL-17 KO mice **(E,F)**. Lesions were blindly scored based on the area with lesions relative to the area of the total visual field. The results are presented as the mean percentage of area with lesions from three mice per group **(G)**. This is one representative result from three different experiments. 100× **(A,C,E)** and 400×**(B,D,F)** magnifications. ^∗^*p* < 0.05.

Considering that the first encounter with the antigen/pathogen by the innate cells will dictate the type and magnitude of the specific immune response, we questioned whether neutrophils were involved in the lesions induced by the vaccination with mc^2^-CMX. To determine this, the subcutaneous lesion at the site of vaccine injection from the C57BL/6 mice was collected and evaluated 15 days after the immunization. **Figure 1** shows that the vaccine induces abscess formation (**Figures 1A,B**). The abscess was primarily composed of a central necrosis surrounded by neutrophils and fibroblasts (**Figure 1B**). The neutrophilic content of the lesion induced by the mc^2^-CMX vaccination suggested that those cells might be important in generating a protective response, which involves specific Th1 and Th17 cells (Junqueira-Kipnis et al., 2013). To address if IL-17 played a role in the recruitment of neutrophils, we used IL-17 KO mice.

IL-17 KO (IL-17ra^-/-^) mice were immunized with mc^2^-CMX and the vaccine lesions were evaluated to assess the role of IL-17 in the innate/adaptive immune response to the live vaccine. The lesions from the IL-17 KO mice were smaller when compared to the lesions from the C57BL/6 mice (observe **Figures 1A,C**). The scoring of the lesion from each mouse evidenced this difference (**Figure 1G**). Because IL-22 has been associated with Th17 induction (Liang et al., 2006) and local inflammation (as well as a synergistic action with IL-17 to induce neutrophil activation; Fouser et al., 2008; Sonnenberg et al., 2010), IL-22 KO mice were also vaccinated. Unexpectedly, the lesions in these mice occupied larger areas than the lesions generated in the IL-17 KO mice (**Figures 1E–G**). Regardless of the mouse genetic background, the lesion induced at the site of vaccination was neutrophilic with an area of central necrosis.

### The Absence of IL-17 Abolishes the Induction of Specific CD4^+^IFN-γ^+^ T Cells in the Spleens of Vaccinated Animals

**FIGURE 2:**
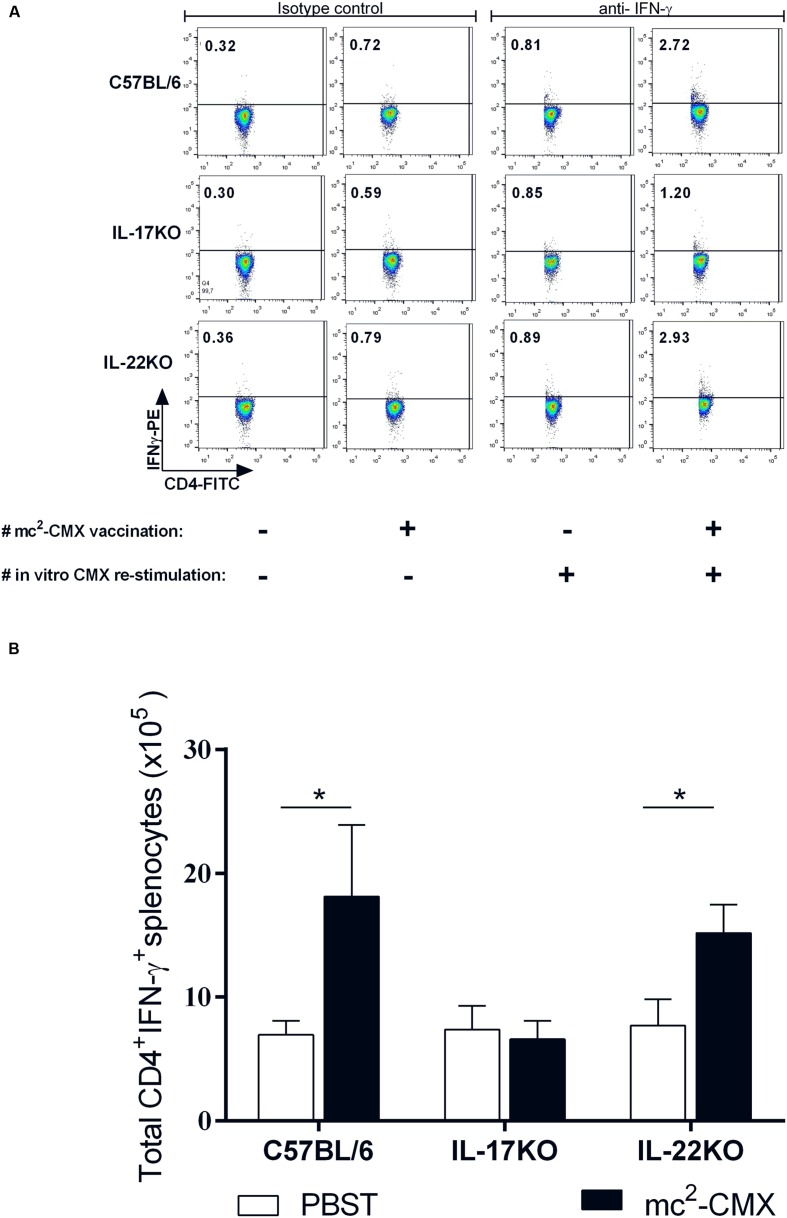
**The IL-17 KO mice do not generate an adequate Th1 anti-CMX specific response after vaccination.** Mice were vaccinated with mc^2^-CMX or with PBST and their splenocytes were *in vitro* re-stimulated with CMX or not. Th1 cells, defined as CD4^+^IFN-γ^+^ T cells were determined after gate analysis using isotype control as reference **(A)**. The total numbers of cells CD4^+^IFN-γ^+^ T splenocytes were determined **(B)**. The results shown represent the mean and standard deviation from a representative experiment. ^∗^*p* < 0.05.

The cytokines and cells that are activated at the site of the infection/vaccination could define the type of specific immune response induced. Therefore, we addressed the question of whether the protective immune response induced by mc^2^-CMX could be maintained in the absence of IL-17 or IL-22. Thus, the response was evaluated in the IL-17 KO and IL-22 KO mice vaccinated with mc^2^-CMX. Fifteen days after immunization, the induced CMX specific gamma interferon-positive CD4 (CD4^+^IFN-γ^+^) T cells were evaluated (**Figure 2A**). While the IL-17 KO mice did not show an increase in the specific CD4^+^IFN-γ^+^ T cells in the spleens of vaccinated animals, the IL-22 KO mice exhibited levels similar to the C57BL/6 vaccinated mice (**Figure 2B**). These results suggest that the Th1- CMX specific response to the vaccine was not adequately generated in the absence of IL-17.

### Vaccination in the Absence of IL-17 Abrogates the Th1-Specific Response and the Protection Induced by mc^2^-CMX Vaccine after *M. tuberculosis* Challenge

**FIGURE 3:**
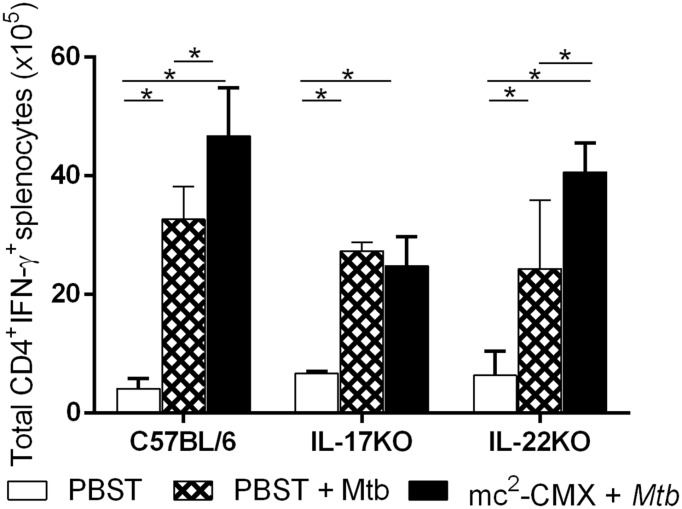
**Absence of Th1 anti-CMX specific recall response among IL-17 KO mice vaccinated and challenged with *M. tuberculosis*.** Mice vaccinated with mc^2^-CMX or with PBST were infected with *M. tuberculosis* and 30 days later their splenocytes were *in vitro* re-stimulated with CMX or not. Th1 cells, defined as CD4^+^IFN-γ^+^ T cells were determined after gate analysis using isotype control as reference. The total numbers of cells CD4^+^IFN-γ^+^ T splenocytes were determined. The results shown represent the mean and standard deviation from a representative experiment. ^∗^*p* < 0.05.

To evaluate if IL-17 and IL-22 were involved in the recall of the immune response generated by the mc^2^-CMX vaccine after *M. tuberculosis* challenge, the wild type, IL-17 KO and IL-22 KO mice were vaccinated with mc^2^-CMX and were infected with *M. tuberculosis* 30 days later. As shown in **Figure 3**, C57BL/6 and IL-22 KO mice vaccinated with mc^2^-CMX exhibited higher total cell number (**Figure 3**) levels of CMX specific CD4^+^IFN-γ^+^ T cells compared to the animals that were only infected with *M. tuberculosis.* Whereas the IL-17 KO mice that were vaccinated and challenged with *M. tuberculosis* presented CD4^+^IFN-γ^+^ response levels similar to that observed with infection of IL-17 unvaccinated mice, which in turn presented response levels similar to the other genetic background mice (compare diamond filled bars of **Figure 3**).

**FIGURE 4:**
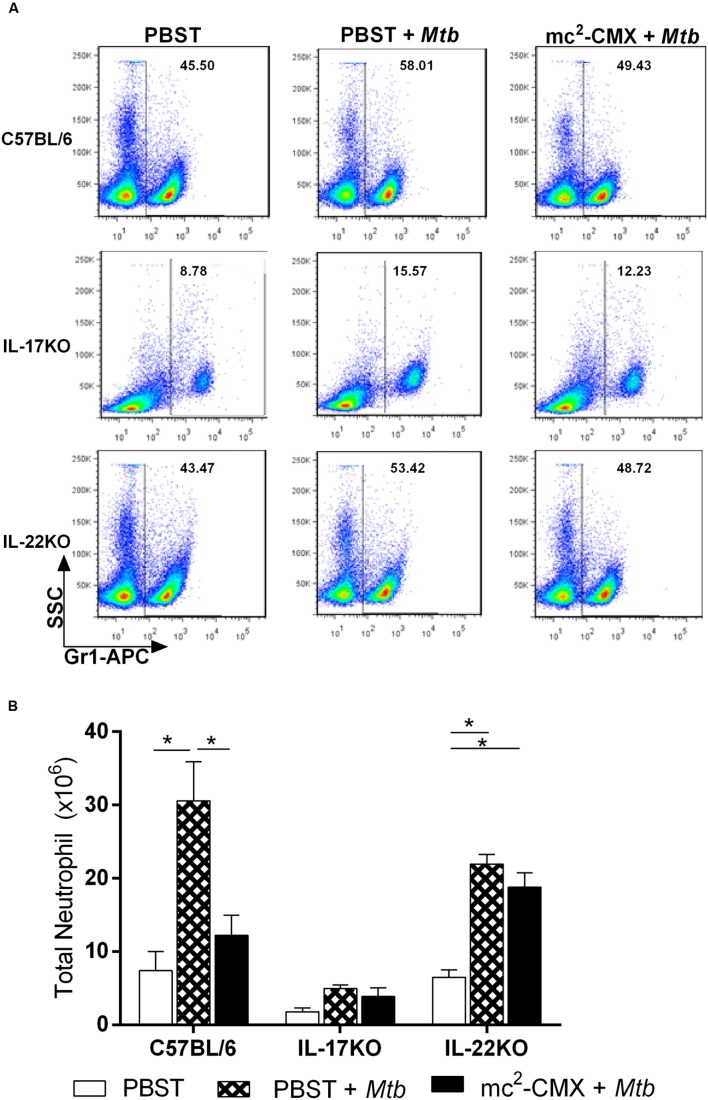
**In the absence of IL-22, neutrophils from the spleens of the vaccinated mice are not modulated in the same manner as in wild type mice.** The C67BL/6, IL-17 KO, and IL-22 KO mice were vaccinated with mc^2^-CMX or PBST and challenged 30 days later with *M. tuberculosis.* **(A)** Representative dot plots showing the neutrophil gating among the different study groups. **(B)** The mean and standard deviation of the total number of neutrophils is presented. The statistical analysis shows comparison between uninfected groups (open bars), vaccinated and *Mtb* infected groups (diamond bars), and *Mtb* infected (solid bars). ^∗^*p* < 0.05.

Neutrophils are also important in *M. tuberculosis* elimination; therefore, we addressed whether the significant influx of Th1 and Th17 cells at 30 days post infection could affect the splenic neutrophil numbers and if vaccination with mc^2^-CMX could modulate this cell population (representative dot plots are shown in **Figure 4A**). Although infection triples the number of neutrophils in the unvaccinated mice, the mc^2^-CMX-vaccinated animals exhibited neutrophils levels that were similar to those of the non-infected mice (**Figure 4B**). Interestingly, vaccination of the IL-22 KO mice did not modulate the number of neutrophils. As expected, due to the lower neutrophil levels exhibited by IL-17 KO mice prior to vaccination, neither vaccination nor *M. tuberculosis* infection increased the number of neutrophils (**Figure 4B**).

**FIGURE 5:**
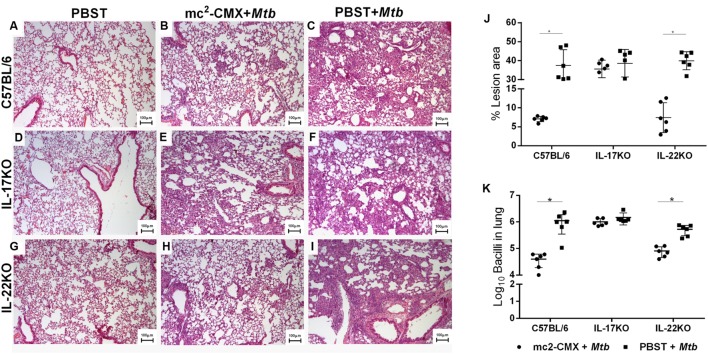
**Inflammatory response to *M. tuberculosis* infection in the lungs of the vaccinated C67BL/6, IL-17 KO, and IL-22 KO mice.** The regular architecture of the lungs from naïve mice **(A,D,G)** is profoundly altered by the inflammatory response induced by *M. tuberculosis* infection **(C,F,I)**. Infection-induced inflammation was reduced when the mice had been previously vaccinated with mc^2^-CMX **(B,E,H)**, although the IL-17 KO mice showed a more discrete reduction **(E)** than the C57BL/6 and IL-22 KO mice **(B,F)**. The lesions of three different lung areas from each animal was scored and calculated as percentage area with lesion **(J)**. Note the absence of lesion reduction [as compared to non-vaccinated and infected animals (circles) and only infected (squares)] in the IL-17 KO group. The mycobacterial load was determined and compared to the non-vaccinated animals (PBST + *Mtb*, squares). **(K)** While the vaccinated C57BL/6 and IL-22 KO mice exhibited approximately one log reduction in the bacterial load compared to the non-vaccinated mice, the vaccinated IL-17 KO mice could not reduce the expected bacterial load. The mean and standard deviation from six mice per group is presented. ^∗^*p* < 0.05.

Because the specific responses induced by the vaccination exhibited different outcomes in the mice with different genetic backgrounds, we decided to verify whether the absence of IL-17 or IL-22 interfered with the lesions and protection induced by the mc^2^-CMX vaccine. The vaccination of all mouse strains prevented the lung inflammatory reactions induced by *M. tuberculosis* infection (**Figure 5J**); nonetheless, the vaccinated C57BL/6 and IL-22 KO mice exhibited fewer parenchyma alterations compared to the IL-17 KO mice (**Figure 5**). The inflammatory lesions of C57BL/6 infected animals were composed by mononuclear cells mainly macrophages and lymphocytes, with some neutrophils diffused throughout the lung parenchyma (**Figure 5C**) while the vaccinated and infected mice presented fewer lesions that were more organized and composed mainly by macrophages and lymphocytes (**Figure 5B**, data not shown). Infected IL-17 KO mice regardless of vaccination status, by other hand, presented diffused inflammatory lesions also composed mainly by macrophages and lymphocytes (**Figures 5E,F**, data not show). The infection of IL-22 KO mice induced huge congestive inflammatory lesions also mainly composed by mononuclear infiltrates and neutrophils (**Figure 5I**, data not show) that were reduced at a lesser extent with mc^2^-CMX vaccination (**Figure 5H**).

The lung bacterial load in mice challenged with *M. tuberculosis*, 30 days after infection, was analyzed. In the absence of IL-17, the mc^2^-CMX vaccine-induced protection was abolished. However, in the absence of IL-22, the vaccine-induced protection was similar to the protection induced in the vaccinated and challenged C57BL/6 mice (**Figure 5K**). Together, the results strengthen the importance of IL-17 in generating a Th1-specific vaccine response after infection. Additionally, in the absence of specific Th1 and Th17 cells (IL-17 KO mice), the vaccine-induced protection was abolished.

### Neutrophils Assist in the Induction of Th1- and Th17-Specific Responses to the mc^2^-CMX Vaccine

**FIGURE 6:**
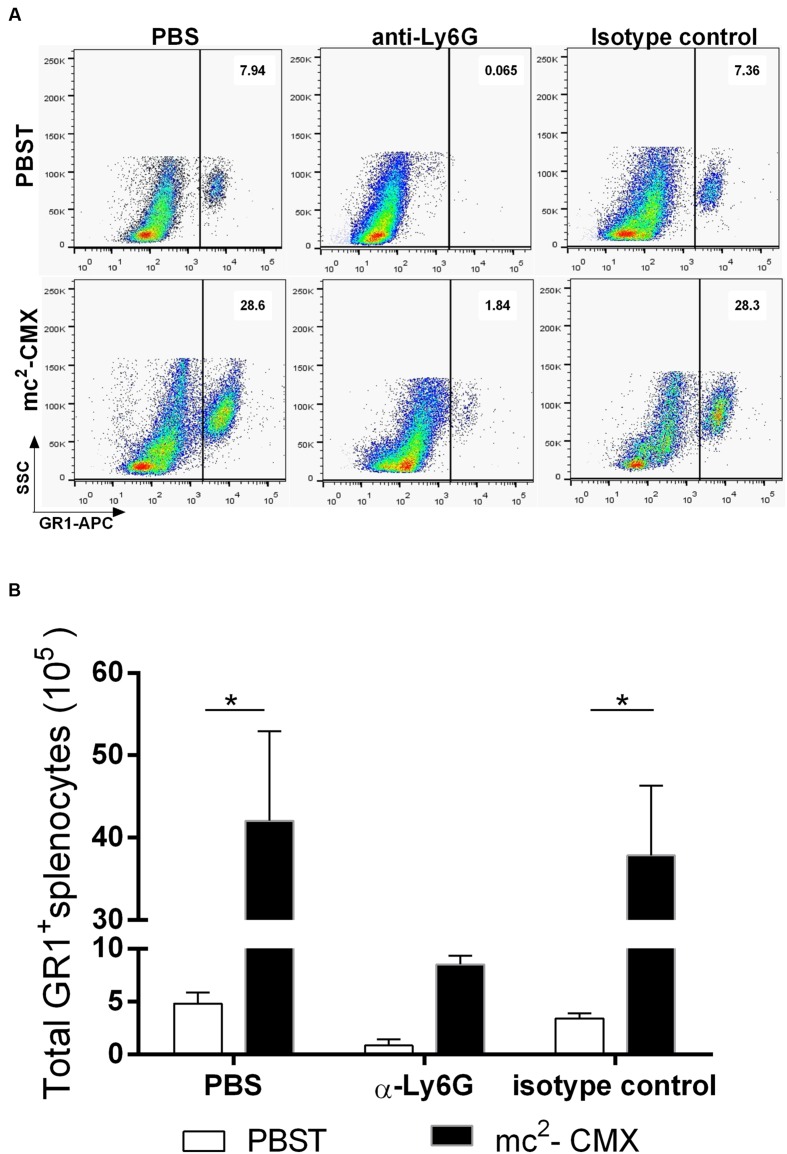
**Neutrophil depletion. (A)** Representative dot plots showing lung neutrophil depletion efficiency. **(B)** The total number of neutrophils in the spleen was calculated and the efficiency of the depletion is shown. The mean and standard deviation from five mice per group is presented. ^∗^*p* < 0.05.

**FIGURE 7:**
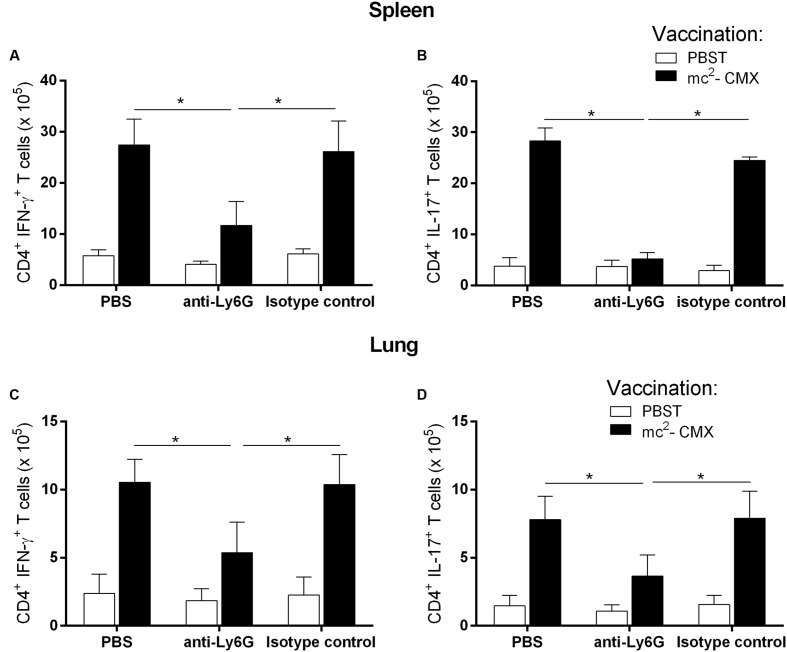
**Neutrophil depletion abrogates the Th1 and Th17 cell induction in the lungs and spleen in response to the mc^2^-CMX vaccination.** C57BL/6 mice treated with anti-Ly6G antibodies (anti-Ly6G) to deplete the neutrophils or with isotype control (isotype control) or with PBS (PBS) were vaccinated with mc^2^-CMX (closed bars) or not vaccinated (PBST, open bars). The increase in the number of Th1 **(A,C)** and Th17 cells **(B,D)** induced by vaccination observed among non-depleted mice (PBST and isotype control) was abrogated in the neutrophil-depleted group. ^∗^*p* < 0.05.

**FIGURE 8:**
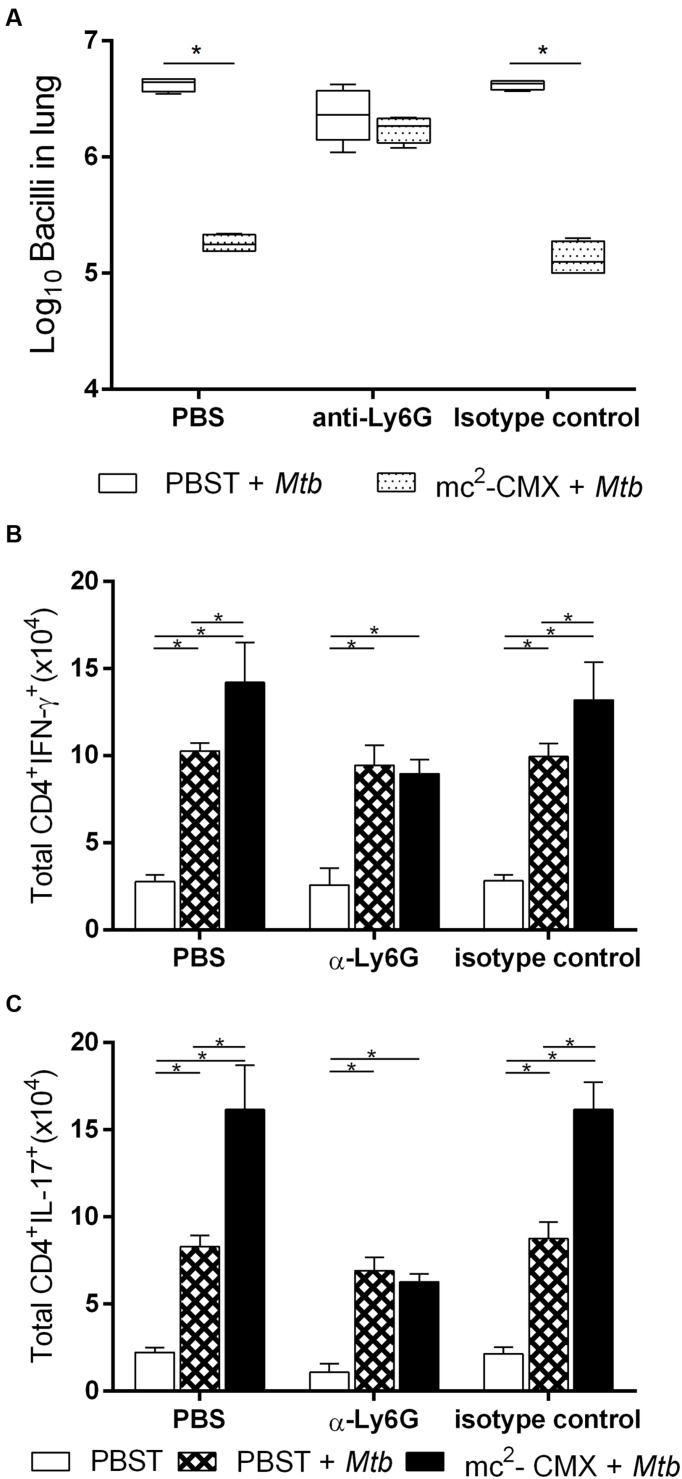
**The absence of neutrophils during mc^2^-CMX vaccination C57BL/6 mice abrogates protection and result in the absence of a Th1 and Th17 recall response.** The C57BL/6 mice were treated with anti-Ly6G to deplete neutrophils or treated with isotype control. Thirty days after *M. tuberculosis* challenge the lung CFU were determined. The vaccine protection efficiency was verified by comparing unvaccinated group (PBST + *Mtb*, open squares) to vaccinated and challenged with *M. tuberculosis* (mc^2^-CMX + *Mtb*, dotted squares) group **(A)**. Three groups of C57BL/6 mice were treated with anti-Ly6G antibodies (α-Ly6G) to deplete the neutrophils, or with isotype antibodies (isotype control) or with PBS (PBS). Within each group a subset was used as naïve uninfected control (PBST, open bars), another as unvaccinated and *M. tuberculosis* challenged animals (PBST + *Mtb*, diamond filed bars) and another groups was vaccinated with mc^2^-CMX and challenged 30 days later with *M. tuberculosis* (mc^2^-CMX + *Mtb*, solid bars). Neutrophil depleted mice were able to generate a Th1 **(B)** and Th17 **(C)** response after *M. tuberculosis* infection (compare diamond filled bars in the different treatment groups). Mice whose neutrophils were depleted during vaccination, when challenged did not show an increase in the Th1 and Th17 response seen among neutrophils sufficient groups (compare diamond filled bars with closed filled bars within each treatment group) ^∗^*p* < 0.05.

The local histological lesions induced by mc^2^-CMX vaccination and the absence of a specific Th1 response in the vaccinated IL-17 KO mice and the absence of neutrophil modulation in the vaccinated and challenged IL-22 KO mice compelled us to raise the question of whether neutrophils were important for the generation of a protective immune response. This question was addressed by treating the wild type mice with an anti-Ly6G antibody or isotype control prior to vaccination and for 21 days after immunization. Neutrophil depletion (**Figures 6A,B**) provoked a significant reduction in the total numbers of CMX specific Th1 (**Figures 7A,C**) and Th17 (**Figures 7B,D**) cells in the lungs and spleen. After *M. tuberculosis* infection, the animals whose neutrophils (Ly6G+) were depleted during vaccination exhibited similar bacterial load compared to that of the non-depleted and non-vaccinated animals (**Figure 8A**).

### Neutrophil Depletion during Vaccination Does Not Interfere with the Immune Response Induced by the Infection with *M. tuberculosis*

In addition to being responsible for the generation of the vaccine CMX specific immune response, neutrophils could have affected the ability of the C57BL/6 mice to respond to *M. tuberculosis*. As shown in **Figure 8**, depletion of neutrophils did not influence the Th1 or Th17 specific cell response (diamonds filled bars, α-Ly6G group infected with *M. tuberculosis* compared with PBS or isotype control treated infected mice). Therefore, early depletion of neutrophils did not interfere with the ability of *M. tuberculosis* infection to induce a Th1 and Th17 response.

On the other hand, the absence of Th1 or Th17 CMX specific recall responses in the lungs of mice that were depleted of neutrophils during vaccination, seen as an increase of the response of vaccinated and infected mice (closed bars) compared to non-vaccinated and infected mice (diamond filled bars), suggest that it may be responsible for the loss of protection induced by the vaccine (**Figure 8**).

## Discussion

This work shows, for the first time, that neutrophils are required for the Th1- and Th17- specific responses generated by a TB vaccine. Additionally, it shows evidence that the IL-17 KO mice vaccinated with mc^2^-CMX have a reduced ability to generate Th1-specific responses and recall these cells after *M. tuberculosis* challenge.

Vaccination with mc^2^-CMX induces increased levels of Th1 and Th17 spleen cells. However, protection against tuberculosis is only achieved when Th1 and Th17 cells that are specific to Ag85, MPT-51, HspX or other *M. tuberculosis* proteins are generated (Sonnenberg et al., 2010; da Costa et al., 2014; Monin et al., 2015). Here, we show that mc^2^-CMX, a recombinant live vaccine vector, induces neutrophilic abscess at the inoculation site that is resolved 30 days after vaccination (**Figure 1**, data not shown). There are lines of evidence indicating that IL-17 and IL-22 induce inflammation and neutrophil migration (Sonnenberg et al., 2010). In this study, the vaccine-induced lesions may reflect a reduction of this function in the absence of IL-17. However, the abscess observed in the IL-22 KO mice did not corroborate this idea, as it was larger than the observed in the wild type mice. IL-22 has been associated with tissue remodeling and as a regulator of the inflammatory reaction (Fouser et al., 2008; Chen et al., 2016), thus the absence of IL-22 could explain the larger lesion observed after mc^2^-CMX vaccination.

Using IL-22 KO mice infected with *M. tuberculosis*, Behrends et al. (2013) showed that this cytokine was not crucial to the control of the infection or the modulation of the specific immune response induced by the infection. However, in humans, IL-22 is directly involved in the specific response to tuberculosis (Scriba et al., 2008). CD4 T cells and granulocytes produce IL-22; however, the effector CD4 T cells from TB patients only increase IL-22 production when stimulated with *M. tuberculosis* proteins *in vitro* (Scriba et al., 2008; Cowan et al., 2012). It is not known whether the IL-22 KO mice exhibit innate and adaptive immune responses to the avirulent *M. smegmatis*, which differ from the response to *M. tuberculosis*. It is important to note that, although they exhibited larger lesion (i.e., abscess), the IL-22 KO mice were able to clear these lesions in less than 1 month (**Figure 1**, data not shown). Here we showed that in the absence of IL-22, the specific Th1 or Th17 responses to mc^2^-CMX vaccination was similar to the response observed among wild type vaccinated mice. Thus, our results add to the knowledge that IL-22 is not crucial for protection as well also to the induction of a protective vaccine against tuberculosis. Nonetheless, the number of splenic neutrophils was not modulated after *M. tuberculosis* infection as observed for C57BL/6 vaccinated mice. This result may indicate that this cytokine is anyhow important for the modulation of the neutrophilic response to *M. tuberculosis* infection and further studies should be done in order to address this fact.

Although controversial, our results show a direct correlation between the induction of Th1-specific responses and the presence of IL-17 in a mouse model of tuberculosis. Our results corroborate those of Khader and Cooper (2008) who suggested that the Th1 and Th17 cells are cross-modulated during infection, and, in the absence of Th17, the protection and the Th1 response induced by a subcutaneous *M. tuberculosis* vaccine (subunit vaccine) were reduced (Khader et al., 2007). Additionally, Umemura et al. (2007) showed that IFN-γ production was reduced following a BCG infection (intratracheal infection) in the absence of Th17 cells. In accordance with our results, these findings imply that both Th1 and Th17 cells were important to induce protection in response to a vaccine against *M. tuberculosis*. In contrast, using a mucosal route of vaccination (subunit vaccine), Gopal et al. (2013) showed that only the Th17-specific cells were crucial to generate protection against *M. tuberculosis* because the vaccinated IFN-γ KO mice showed some level of protection that was abolished after IL-17 depletion. This scenario suggests that the vaccine type and its components may be important for defining the specific immune response induced by a vaccine, as both authors used ESAT-6 as subunit vaccine against *M. tuberculosis* but used different routes of vaccination and different adjuvants. Our results and those of others confirmed that subcutaneous vaccination induces both Th1- and Th17-specific responses that are important for the protection of the mice against *M. tuberculosis* and that the presence of IL-17 is important for the induction of Th1 responses.

In this study, it is clear the role of specific Th1 and Th17 cells in the protection induced by mc^2^-CMX vaccine, however, it was not addressed if neutrophil depletion alters the CD8 T lymphocytes activation or mycobactericidal function, fact that could have contributed to the loss of the protection (Behar, 2013; Lin and Flynn, 2015). In this sense neutrophils could be important for DC crosspriming for *M. tuberculosis* antigens that would help its mycobactericidal function during the post vaccinal challenge.

Several studies have shown that the protective immune response to bacterial pathogens involves a Th17-specific response. For instance, the protection induced by vaccines for *Bordetella pertussis* (PW vaccine), *Streptococcus pneumonia* (WCV vaccine), and *Staphylococcus aureus* (TSST-1 vaccine) was reduced when the animals were treated with anti-IL-17 antibodies or when IL-17ra KO or IL-17 KO mice were used (Lu et al., 2008; Ross et al., 2013; Narita et al., 2015).

Several mechanisms might be involved in the generation of the Th1- and Th17-specific immune responses by some *M. tuberculosis* vaccines. In this study, we show, for the first time, a direct role for neutrophils. The antimicrobial functions of neutrophils are very well known, and their deficiency causes severe bacterial and fungal infections (Mantovani et al., 2011). For example, in tuberculosis, neutrophils form neutrophil extracellular traps (NETs) that have been associated with macrophage activation (Braian et al., 2013), and neutrophils elastases are important in the control of mycobacterial growth (Steinwede et al., 2012). In addition to this innate immune response, neutrophils also assist in NK, B, and DC cell activation (Jaeger et al., 2012; Schuster et al., 2013). Here, we hypothesized that because neutrophils are clearly involved in the response to this live vaccine and the reduction or absence of neutrophils reduces both the Th1- and Th17-CMX specific recall responses, they might be responsible for activating DCs to induce both cells. This hypothesis is corroborated by the fact that the DCs that ingest infected neutrophils are better activated than the DCs that acquire mycobacterial antigens in the absence of neutrophils (Nakae et al., 2002). Rather, someone may argue that IL-17 KO mice are not more susceptible to *M. tuberculosis* infection, although showed a reduced neutrophilic response after the challenge when compared to the wild type mice, and therefore neutrophils are not important. Contrary to this observation, the results presented here show the importance of these cells for the generation of specific Th1 and Th17 cells induced by a live vector vaccine against TB and do not intend to show its importance on the infection.

The vaccine design used in this study has been shown previously to generate specific Th1 and Th17 responses (against CMX) that culminated with better protection than BCG against an intravenous *M. tuberculosis* challenge (Junqueira-Kipnis et al., 2013). Our results and those of others confirmed that subcutaneous vaccination induces both Th1- and Th17-specific responses that are important for the protection of the mice against *M. tuberculosis* (Gopal et al., 2013). Here we show that the presence of IL-17 and neutrophils are important for the induction of a CMX Th1-specific response. Someone may reason that intravenous *M. tuberculosis* infection may not be the natural route of infection, as tuberculosis is primarily a pulmonary disease, nonetheless these results are in agreement with the literature including those studies that use aerosol mouse model of infection. Though, it will be interesting in the future to address if the neutrophils are also needed for other vaccines as well as using different routes of infection.

## Conclusion

This work shows for the first time that neutrophils are important for the specific induction of Th1 and Th17 cells in response to a live vaccine against *M. tuberculosis*.

## Author Contributions

AK and AJ-K conceived the experiments. MT and FO developed the experiments. MT and AJ-K draft the manuscript. All authors read, critically revised and approved the manuscript.

## Conflict of Interest Statement

The authors declare that the research was conducted in the absence of any commercial or financial relationships that could be construed as a potential conflict of interest.
